# Enhanced Thermoelectric Efficiency of Porous Silicene Nanoribbons

**DOI:** 10.1038/srep09514

**Published:** 2015-03-30

**Authors:** Hatef Sadeghi, Sara Sangtarash, Colin J. Lambert

**Affiliations:** 1 Quantum Technology Centre, Lancaster University, LA1 4YB Lancaster, UK

## Abstract

There is a critical need to attain new sustainable materials for direct upgrade of waste heat to electrical energy via the thermoelectric effect. Here we demonstrate that the thermoelectric performance of silicene nanoribbons can be improved dramatically by introducing nanopores and tuning the Fermi energy. We predict that values of electronic thermoelectric figure of merit *ZT_e_* up to 160 are achievable, provided the Fermi energy is located approximately 100 *meV* above the charge neutrality point. Including the effect of phonons yields a value for the full figure of merit of *ZT = * 3.5. Furthermore the sign of the thermopower *S* can be varied with achievable values as high as *S* = +/− 500 μV/K. As a method of tuning the Fermi energy, we analyse the effect of doping the silicene with either a strong electron donor (TTF) or a strong electron acceptor (TCNQ) and demonstrate that adsorbed layers of the former increases *ZT_e_* to a value of 3.1, which is insensitive to temperature over the range 100 K – 400 K. This combination of a high, temperature-insensitive *ZT_e_*, and the ability to choose the sign of the thermopower identifies nanoporous silicene as an ideal thermoelectric material with the potential for unprecedented performance.

In the recent years, the challenge of removing heat from nanoelectronic devices^1^ and thermoelectrically converting waste heat into electricity has attracted huge scientific interest^2^, not only due to the questions posed for fundamental science, but also because thermoelectric energy conversion is an essential requirement for the next generation of nanoscale electronic, optoelectronic and photonic devices^2^. The efficiency of a thermoelectric device and material is determined by its thermoelectric figure of merit (*ZT*) defined as:
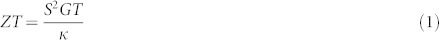
where *S* is the Seebeck coefficient (thermopower), *G* is the electrical conductance, *T* the temperature and *κ* the thermal conductance given by *κ* = *κ_e_* + *κ_p_*, where *κ_e_* (*κ_p_*) is the electronic (phononic) contribution to *κ*^3^. Clearly *ZT* could be enhanced by increasing the power factor (*S*^*2*^*GT*) or decreasing the thermal conductance and therefore a high-performance thermoelectric material should possess a large Seebeck coefficient and electrical conductance and simultaneously a low thermal conductance. The search for new materials with enhanced thermal properties continues to intensify, because these factors are correlated and increasing *ZT* to values greater than unity requires a delicate optimisation of several material properties.

One promising approach has been to reduce the contribution *κ_p_* of parasitic phonons by nanostructuring materials^4^. Although acoustic phonons are the main heat carriers in bulk crystals, nanostructures can exhibit significantly lower phonon thermal conductances *κ_p_*, due to increased phonon boundary scattering, changes in the phonon density of states and modified phonon dispersion in low-dimensional materials^2^. The nanostructuring of materials is also a promising route to increasing the power factor, because it can lead to sharp features in the electronic density of states and the transmission coefficient *T(E)* describing the propagation of electrons of energy *E* through a device. It is well known that the thermopower *S* is controlled by the asymmetry of *T(E)* at the Fermi energy *E_F_* and therefore if *E_F_* is close to such an asymmetry, both *S* and *ZT* will be enhanced. Unfortunately, asymmetries, even if they occur, are not necessarily located near *E_F_* and therefore a method of tuning them is required. In a nanoscale device, one could of course consider introducing a third gate electrode to control such features, but in practice this costs energy and does not solve the problem of designing a new material.

The above considerations suggest that an effective strategy for enhancing thermoelectric properties should 1) start from a parent material with low intrinsic thermal conductance, 2) nanostructure the material to further reduce the thermal conductance, 3) implement additional nanostructuring to introduce sharp features in *T(E)* and 4) chemically modify the material to move these sharp features towards the Fermi energy. In this paper our aim is to demonstrate that silicene, a new counterpart of graphene is an ideal material for implementing the four key elements of this strategy, because it is not only CMOS-compatible, but also it possesses a low intrinsic thermal conductance, which can be further reduced by nanostructuring the material to form nanoribbons.

Since silicon is the most common material used in the electronics industry, it is highly desirable to utilise silicon-compatible materials for thermoelectric energy conversion. Bulk silicon has a very low *ZT* (≈0.01) and therefore as a first step in a strategy for enhancing thermoelectric performance it is natural to focus on silicene, which is a recently-observed one-atom-thick crystalline form of silicon atoms arranged in a slightly buckled honeycomb lattice structure^5^,^6^,^7^,^8^,^9^,^10^,^11^,^12^. Silicene nanoribbons have been synthesised on silver (111)^7^,^13^,^14^,^15^,^16^,^17^,^18^,^19^,^20^,^21^,^22^,^23^,^24^,^25^, gold (110)^26^, iridium (111)^27^ and the zirconium diboride (0001)^28^,^29^ substrates and are predicted to be stable on non-metallic substrates^30^. Calculations of thermoelectric properties of armchair and zigzag silicene nanoribbons with and without hydrogen-passivated edges suggest that this material may be attractive for thermoelectric devices^31^,^32^,^33^,^34^, because the thermal conductivity of 2D silicene is predicted to be much smaller than bulk silicon and its counterpart graphene^35^, with only ~10% of the total phononic thermal conductivity being due to the out-of-plane acoustic phonons^36^,^37^. Both of these desirable features arise from the presence of small buckling, which breaks the reflection symmetry of the structure^35^,^36^. Further reductions in the phonon contribution arise from additional nanostructuring. For example the thermal conductivity of a silicene nanosheet 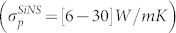
31,35,36,37 reduces to 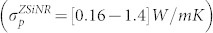 in zigzag silicene nanoribbons^32^,^33^,^34^. This could be potentially even lower in porous silicene, since the lattice thermal conductivity is reduced in nanoporous semiconductors^38^ such as nanoporous Bi^39^, Ge^40^, graphene^41^,^42^,^43^, Bi_2_Te_3_^44^ and SiGe^45^. Moreover, it is been shown that placing nanopores in bulk silicon greatly reduces the thermal conductivity and enhances *ZT* by the factor of two^46^.

Since the low phonon thermal conductance of silicene is well established and therefore elements 1) and 2) of the above strategy are satisfied, in this paper we focus steps 3) and 4) and demonstrate that they can be achieved by inserting nanopores into silicene nanoribbons, whose edges are terminated by hydrogen or oxygen and by introducing adsorbates to tune the position of features in *T*(*E*) relative to *E_F_*. In view of the low value of *κ_p_*, we will focus primarily on the electron contribution in thermoelectric figure of merit *ZT_e_* (obtained by setting κ_p_ = 0 in equation 1) and show that for ribbons containing nanopores with hydrogen-passivated edges, *ZT_e_* could be enhanced to values as high as 160 at room temperature by creating sharp features in the *T(E)* and controlling the Fermi energy or by introducing adsorbates such as Tetrathiafulvalene (TTF) and Tetracyanoquinodimethane (TCNQ) onto the silicene surface. We will then show that by including the *κ_p_* of silicene nanoribbons, a high value of the full *ZT* of order 3.5 is achievable.

## Results and Discussion

**Figure 1:**
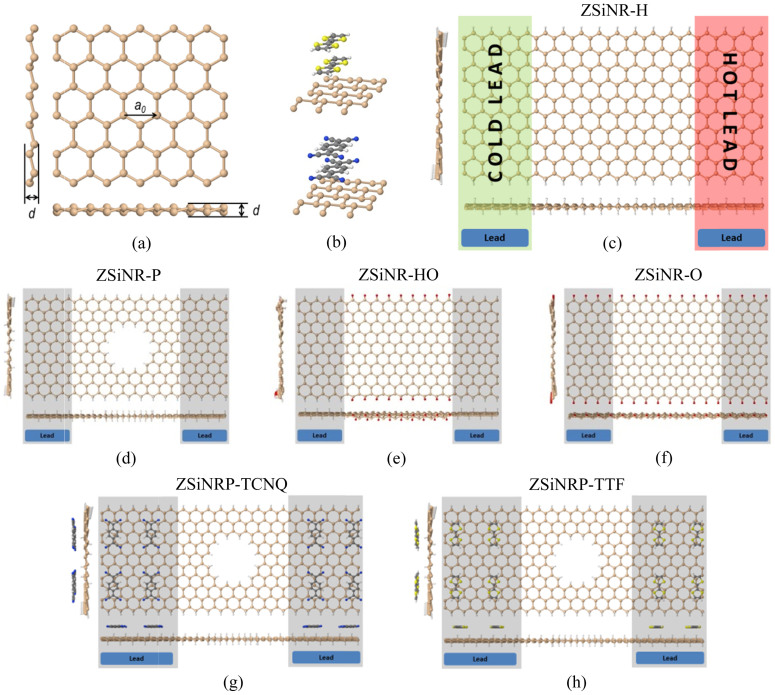
Sketch of the molecular structure of the Silicene and its alloys. (a) silicene molecular structure, (b) a *TTF* (top) and *TCNQ* (bottom) doped silicene ribbon with two layers of dopant, (c) silicene monolayer ribbon with hydrogen terminated edges (ZSiNR-H), (d) silicene monolayer containing a nanopore (ZSiNR-P). All edges are terminated with hydrogen. (e) silicene monolayer ribbon with an oxygen terminated scattering region and hydrogen terminated electrodes (ZSiNR-HO), (f) silicene monolayer ribbon with oxygen terminated edges (ZSiNR-O), (g) as for (d), but with adsorbed Tetracyanoquinodimethane (TCNQ) and (h) as for (d), but with adsorbed Tetrathiafulvalene (TTF).

We have calculated the electronic contribution to the thermal conductance, the Seebeck and Peltier coefficients and ZT*_e_* for the structures shown in Figure 1c–h. The optimized lattice constant (*a_0_*) and buckling (*d*) of the silicene nanoribbon shown in Figure 1a are found to be 3.6 Å and 0.53 Å, respectively, similar to that reported elsewhere^10^,^32^,^47^. The engineered silicene nanoribbons shown in Figure 1 include: a zigzag monolayer silicene nanoribbon with hydrogen terminated edges (ZSiNR-H, Figure 1c), a zigzag monolayer silicene nanoribbon containing a nanopore with all edges terminated by hydrogen (ZSiNR-P, Figure 1d), a zigzag monolayer silicene nanoribbon with a central region containing oxygen terminated edges, connected to hydrogen-terminated leads (ZSiNR-HO, Figure 1e), and a zigzag monolayer silicene nanoribbon with oxygen terminated edges (ZSiNR-O, Figure 1f). The nanoribbons length and width in all cases are almost equal (*L* ≈ 6 *nm* (scattering region ≈ 3.48 *nm*), *W* ≈ 3 *nm*) and the pore diameter is ≈1.3 *nm*. We shall find that a key strategy for improving the thermoelectric performance of these structures involves tuning the Fermi energy (*E_F_*). As an example of how this could be achieved, we investigated the effect of introducing adsorbates onto the surface of the ZSiNR-P hydrogen-terminated nanoribbon, as shown in Figures 1f and 1g. The latter show examples of such functionalised ribbons containing adsorbed TTF (a strong electron donor) and TCNQ (a strong electron acceptor), which form charge-transfer complexes with the silicene.

### Silicene nanoribbons and nanopores

**Figure 2:**
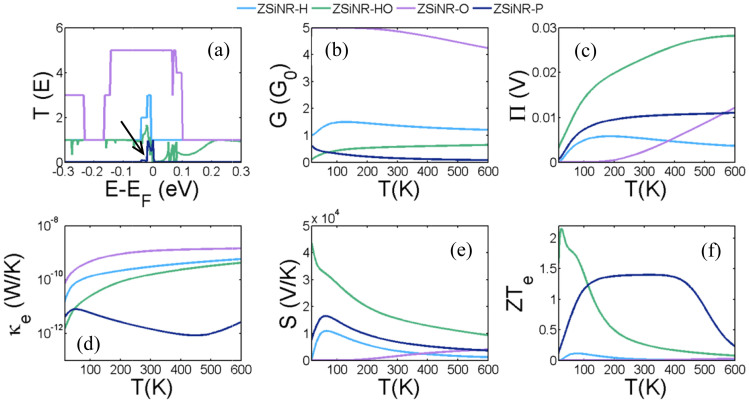
Thermoelectric properties of the ZSiNR-H, ZSiNR-HO, ZSiNR-O and ZSiNR-P. (a) Transmission coefficient *T(E)* (see Supplementary Information (SI) for a magnified version of this figure), (b, c) electrical and thermal conductance (G, κ), (d, e) Peltier (Π) and Seebeck (S) coefficients and (f) figure of merit (*ZT_e_*) as a function of temperature in the ZSiNR-H, ZSiNR-HO, ZSiNR-O and ZSiNR-P.

For the structures (c) to (f) of Figure 1, Figure 2 shows results for electrical conductance *G*, the electronic contribution of the thermal conductance *κ_e_*, the thermopower *S* and the Peltier coefficient *Π* of the junction as a function of the temperature (*T*). All of these properties are obtained from the energy (*E*) dependence of the electron transmission coefficient *T(E)*, shown in Figure 2a.

Figure 2 shows that the silicene nanoribbons ZSiNR-H, ZSiNR-HO and ZSiNR-O possess high electronic thermal conductances and low thermopowers and consequently their figures of merit (*ZT_e_*) are low. However, the results for the silicene monolayer nanoribbon containing a nanopore (ZSiNR-P) show that *ZT_e_* is significantly improved by placing a hole in the ribbon. This arises because the densities of states of all four structures possess sharp peaks around the Fermi energy *E_F_* due to band bending in the corner of the *k*-space in silicene band structure, which introduces a sharp feature (indicated by an arrow in Figure 2a) in *T(E)* near *E_F_*. Furthermore the *T(E)* of ZSiNR-P possess the desirable feature that *T(E)* is almost zero on either side of the peak. This feature is associated with edges states, which is why it is sensitive to the chemical nature of the edge terminations. These edge states are a well-known feature of nanoribbons with zigzag edges^48^,^49^,^50^ and lead to a significant improvement in the thermopower (Figure 2e) as well as a reduction in the thermal conductance (Figure 2d). Edge states have been predicted earlier theoretically^51^ and observed experimentally^52^,^53^,^54^ in graphene nanoribbons. By comparing ZSiNR-P with ZSiNR-H or by comparing ZSiNR-HO with either ZSiNR-P or ZSiNR-O, Figure 2 demonstrates the general trend that introducing scattering reduces both the electrical and thermal conductances and simultaneously increases the thermopower. Figure 2 also reveals the attractive property that ZSiNR-P possesses the highest room-temperature *ZTe* (of order 1.4) and that this high thermoelectric efficiency is preserved over a wide range of temperature from 100 K to 500 K.

**Figure 3:**
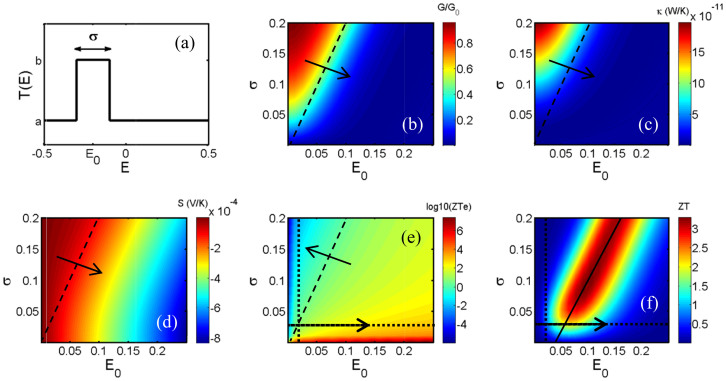
Thermoelectric properties of a system with Delta function like transmission coefficient *T*(*E*). (a) Delta transmission coefficient with the width of *σ* and mean of *E_0_* and a = 0 and b = 1 and corresponding (b) conductance (*G/G_0_*), (c) electronic thermal conductance (*κ_e_*), (d) Seebeck coefficient (*S*), (e) logarithm of electronic *ZT* (*log_10_*(*ZT_e_*)) and (f) total *ZT* (with phononic and electronic contribution) as a function of σ and *E_0_* in the room temperature. *E_0_* represents the position of the peak in the transmission function relative to the Fermi energy *E_F_ = 0*.

To highlight the role of sharp features near *E_F_* in the transmission coefficients of Figure 2a, we investigate a simple model for which *T*(*E*) = *a* + *b* for 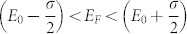 and *T*(*E*) = *a* for *E_F_* outside this range. As shown in Figure 3a, possesses a rectangular peak of height *b*, width σ, located at an energy *E_0_* relative to the Fermi energy *E_F_* = 0 *eV*. The peak is superposed on a constant background of height *a*. Crudely, on the scale of *k_B_T* and for relevant values of *E_0_*, the transmission coefficient of ZSiNR-P corresponds to the case *b* = *1*, *a* = *0*, ZSiNR-O corresponds to *a = b* = *5* (ie no peak), whereas ZSiNR-H corresponds to *a* = *1*, *b = 3*.

For the case of ZSiNR-P, where *b* = *1*, *a* = *0*, Figures 3b–e show the conductance (*G/G_0_*), electronic thermal conductance (*κ_e_*), Seebeck coefficient (*S*) and the logarithm of electronic figure of merit (*ZT_e_*) as a function of *σ* and *E_0_* at room temperature. As shown in Figure 3e (right hand side of horizontal dashed line), *ZT_e_* could increase up to 10^6^ provided the width *σ* is sufficiently small. However, for a broad transmission peak close to Fermi energy one obtains a very low electronic thermoelectric figure of merit down to ~10^−5^ (left hand side of dashed line). This is mainly due to reduction of electronic thermal conductance (Figure 3c), because the thermopower decreases more slowly (Figure 3d) than the electronic thermal conductance.

**Figure 4:**
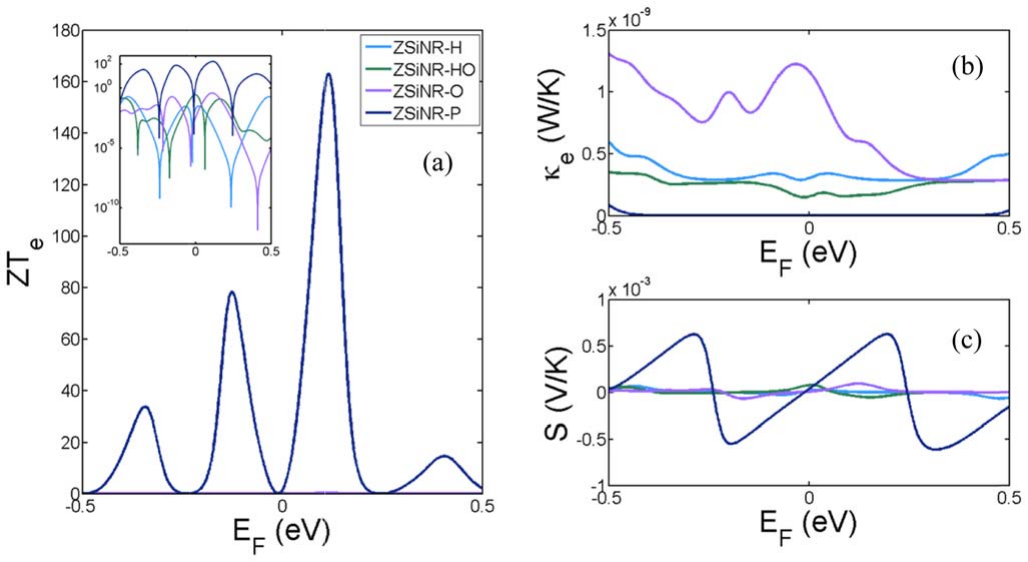
Room temperature thermal properties of the ZSiNR-H, ZSiNR-HO, ZSiNR-O and ZSiNR-P in different Fermi energies *E_F_*. (a) The variation of room-temperature values of *ZT_e_*, (inset is *ZT_e_* on the logarithmic scale), (b) electronic thermal conductance *κ_e_* and (c) Seebeck coefficient *S* as a function of *E_F_* for ZSiNR-H, ZSiNR-HO, ZSiNR-O and ZSiNR-P. The main part of fig. 4a shows *ZT_e_* for ZSiNR-P on a linear scale.

The above simple model and the results of Figure 2 demonstrate that the thermoelectric performance of silicene nanoribbons is improved by the introduction of nanopores. To demonstrate that further dramatic improvements are available if the Fermi energy is tuned by an external gate, figure 4 shows results for *ZT_e_*, *S* and *κ_e_* as a function of *E_F_*, obtained from the exact transmission curves *T(E)* of Figure 1a. This demonstrates that at room temperature, the electronic contribution to *ZT_e_* can be hugely enhanced by varying the Fermi energy by as little as 100 *meV*, with achievable values as high as 160. Furthermore the sign of the thermopower can be selected by such tuning, with achievable values as high as *S* = +/− 500 μV/K.

This improvement could be achieved by electrostatically-gating the nanopore-containing ribbon. For the purpose of improving intrinsic material performance, we demonstrate below that a Fermi energy shift can also be realised by introducing donor or acceptors onto the surface of the silicene nanoribbon. However before considering the effect of doping, we examine the effect of phonons, since *ZT_e_* is obtained by neglecting the phononic contribution (*κ_p_*) to the thermal conductance in equation (1). This is a reasonable approximation to the full figure of merit *ZT* provided the electronic thermal conductance (*κ_e_*) is higher than phononic thermal conductance. However large values of *ZT_e_* correspond to the opposite limit of low values of *κ_e_*, where *κ_p_* > *κ_e_*. In this limit, one needs to consider only the phononic contribution and consequently the denominator of equation (1) becomes a constant, independent of *σ* and *E_0_*.

To estimate the *κ_p_* for the structure of figure 1d, we note that for a perfect zigzag silicene nanoribbon of width equal to the bridging sections of silicene above and below the pore of Figure 1d is predicted to have a phononic thermal conductance *κ_p_* of less than 6 × 10^−11^ W/K^32^. This value contrasts markedly with graphene, whose thermal conductance is predicted to be a factor of at least 50 times higher^36^. As discussed in the introduction, boundary scattering by nanopores can lead to a further order of magnitude reduction in *κ_p_* and therefore a value of *κ_p_* on the scale of ~10^−12^ W/K would seem reasonable for the structure of Figure 1d. For the model *T(E)* of Figure 3a, Figure 3f shows the full *ZT* when the thermal conductance is dominated by phonons, with a high thermal conductance of *κ_p_* = 2 × 10^−11^ W/K. This demonstrates that even in the most unfavourable scenario, the full *ZT* could achieve a value as high as 3.5 for values of *σ* and *E_0_* lying in the red region of Figure 4f (where *σ* ~ 2*E_0_* − 0.1). The transmission coefficient of the silicene nanopore (ZSiNR-P) shown in Figure 2a could be approximately modelled by *b* = 1, *a* = 0 with the width of *σ* = 0.025 and a mean of *E_0_ = * 0.015 around the Fermi energy. The crossover of dotted lines in Figure 3e shows the model *ZT_e_* at room temperature, which is in good agreement with the value of *ZT_e_* obtained from the more accurate *T(E)* shown in Figure 2f. By increasing *E_0_*, Figure 3f suggests that values of the full figure of merit as high as *ZT* = 3.5 are possible. On the other hand (see SI) for the ZSiNR-O (*a = b* = *5*) and ZSiNR-H (*a* = *1*, *b = 3*), the presence of a non-zero background suppresses the achievable values of *ZT*. For this reason, we now focus on tuning the properties of the H-terminated nanopore and demonstrate that high values of *ZT_e_* can be achieved by doping the surface of the silicene.

### Adsorbate-functionalized silicene

For the purpose of improving intrinsic material performance, a method of shifting *E_F_* by doping the silicene nanoribbon is needed. Since covalent bonding of dopants to the nanoribbons may adversely affect electronic properties, we now consider tuning the Fermi energy by adsorbing planar molecules which interact with the surface only through weak pi-pi interactions. The first adsorbed molecule we study is Tetrathiafulvalene (TTF) which is a strong donor and the second is Tetracyanoquinodimethane (TCNQ), which is a strong acceptor^55^.

**Figure 5:**
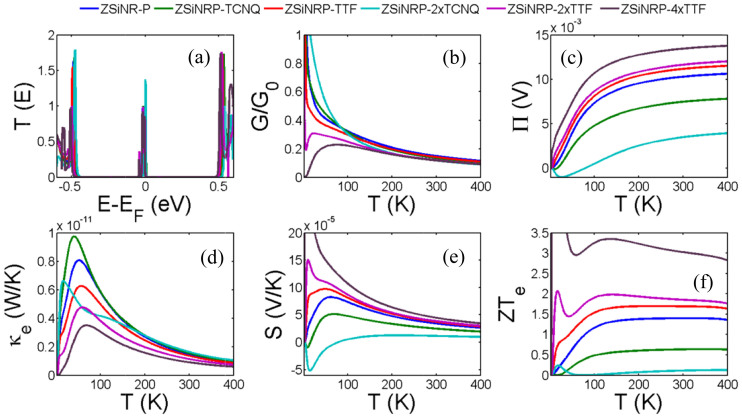
Thermoelectric properties of the intrinsic and doped ZSiNR-P with various dopant and concentrations. (a) Transmission coefficient *T(E)* (see SI for a magnified version of this figure), (b, c) electrical and thermal conductance (G, κ), (d, e) Peltier (Π) and Seebeck (S) coefficients and (f) figure of merit as a function of temperature. Results are shown for a monolayer silicene nanopore (ZSiNR-P) with a perfect zigzag silicene ribbon electrodes, a monolayer silicene nanopore with TCNQ functionalized zigzag silicene nanoribbon electrodes with low concentration of TCNQ (ZSiNRP-TCNQ) and with higher concentration of TCNQ (ZSiNRP-2×TCNQ), a monolayer silicene nanopore with TTF functionalized zigzag silicene ribbon electrodes with low concentration of TTF (ZSiNRP-TTF) and with higher concentrations of TTF (ZSiNRP-2×TTF and ZSiNRP-4×TTF).

**Figure 6:**
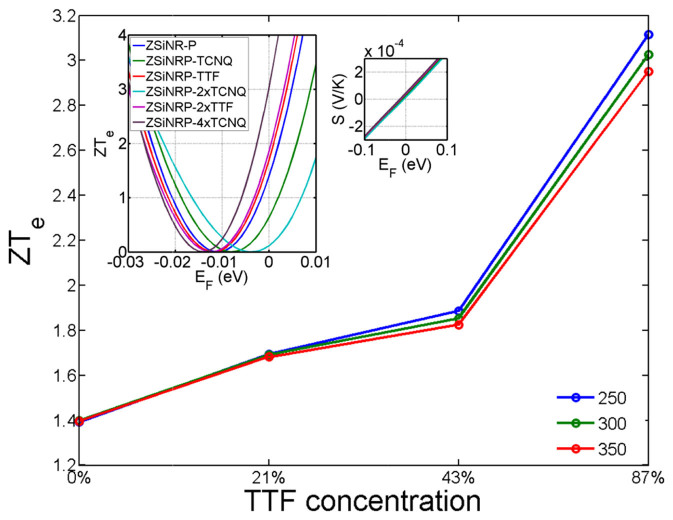
The effect of the dopant on the thermoelectric figure of merit. Thermoelectric figure of merit (*ZT_e_*) in *E_F_* = 0 for ZSiNR-P doped with 0%, 21%, 43% and 87% TTF in *T* = 250 K, 300 K and 350 K. inset: The variation of room-temperature (*T* = 300 K) values of *ZT_e_* as a function of *E_F_* in intrinsic and doped ZSiNR-P with 21% TTF (ZSiNRP-TTF), with 43% TTF (ZSiNRP-2×TTF), and with 87% TTF (ZSiNRP-4×TTF).

In the presence of adsorbates, calculations were performed by relaxing the adsorbates to find their minimum-energy states on the surface of the silicene (see methods), which yielded an optimized distance between the silicene and TCNQ of 3.1 Å and 3.54 Å for TTF. For the lowest dopant concentrations, we place a monolayer or sub-monolayer of TTF or TCNQ on the surface of the electrodes of ZSiNR-P, as shown in Figures 1g and 1h, whereas for the highest concentration, two layers above and below the silicene are included, as shown in Figure 1b. Figure 5 shows that TTF on silicene simultaneously increases the Seebeck coefficient, decreases the thermal conductance and increases *ZT_e_* from an undoped value of 1.4 to 1.7, 1.85 and 3.1 with increasing concentration from 21% to 43% and 87%, where the concentration of adsorbate is defined as the ratio of the number of TTF atoms to the number of silicene nanoribbon atoms. The inset of Figure 6 shows the variation of *ZT_e_* with *E_F_* for different concentrations of TTF and reveals that an 87% coverage produces a Fermi energy shift of approximately 3 *meV*. The transmission just around the Fermi energy is shown in Figure S2b of SI.

In summary, we have developed a new strategy for improving the thermoelectric performance of silicene-based nanoribbons by investigating the effect of introducing nanopores and varying their edge termination. We have demonstrated that the thermopower and electronic thermoelectric figure of merit *ZT_e_* can be improved by introducing nanopores and tuning their Fermi energy. By shifting the Fermi energy by approximately 100 *meV* from the charge-neutrality point, we predict that huge values of *ZT_e_* up to 160 are accessible and that the sign of the thermopower can be varied with achievable values as high as *S* = +/− 500 μV/K. As a method of tuning the Fermi energy, we analysed the effect of doping the silicene with either a strong electron donor (TTF) or a strong electron acceptor (TCNQ) and demonstrated that doping by the former tended to decrease the value of *ZT_e_*, because the Fermi energy shift was in an unfavourable direction. On the other hand, we found that doping with TTF increased the room-temperature value to *ZT_e_* to 3.1 and that this value is insensitive to temperature over the range 100 K – 400 K. This combination of a high temperature-insensitive *ZT_e_*, and the fact that the low phonon thermal conductance renders *ZT_e_* comparable with the full figure of merit *ZT*, identifies nanoporous silicene as an ideal thermoelectric material with the potential for unprecedented performance.

## Methods

To find the optimized geometry and ground state Hamiltonian of the structures of interest, we employed the SIESTA^56^ implementation of Density Functional Theory (DFT) using the generalized gradient approximation (GGA) of the exchange and correlation functional with the Perdew-Burke-Ernzerhof parameterization (PBE)^57^ a double zeta polarized basis set, a real-space grid defined with a plane wave cut-off energy of 250 Ry and a maximum force tolerance of 40 meV/Ang. The calculation with VDW-DF exchange and correlation functional with BH parameterization was also carried out to check the GGA-PBE result in some cases. The BH is the same as DRSLL with some modification^58^. From the converged DFT calculation, the underlying mean-field Hamiltonian was combined with our transport code, GOLLUM^59^ which is an implementation of the non-equilibrium Green's function (NEGF) method. This yields the transmission coefficient *T*(*E*) for electrons of energy *E* (passing from the source to the drain) via the relation
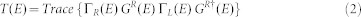
In this expression, *Γ_L,R_*(*E*) = *i* (Σ*_L,R_*(*E*) − Σ*_L,R_*^†^(*E*)) describes the level broadening due to the coupling between left (L) and right (R) electrodes and the central scattering region, Σ*_L,R_*(*E*) are the retarded self-energies associated with this coupling and *G^R^* = (*ES* − *H* −Σ*_L_* − Σ*_R_*)^−1^ is the retarded Green's function, where *H* is the Hamiltonian and *S* is overlap matrix (both of them obtained from SIESTA). Thermal properties such as the electrical conductance *G*(*T*), the electronic contribution of the thermal conductance *κ_e_*(*T*), the thermo-power *S*(*T*) and the Peltier coefficient *Π*(*T*) of the junction as a function of the temperature are given by^59^:
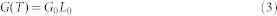

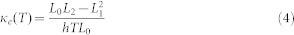

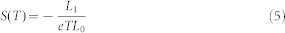

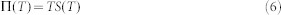


where
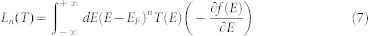
and *T*(*E*) is the transmission coefficient, *f*(*E*) is the Fermi-Dirac probability distribution function (*f*(*E*) = (1 + *exp* (*E* − *E_F_*/*k_B_T*))^−1^), *T* is the temperature, *E_F_* is the Fermi energy, *G*_0_ = 2*e*^2^/*h* is the conductance quantum, *e* is electron charge and *h* is the Planck's constant. From these expressions, the electronic contribution to the figure of merit *ZT_e_* is:
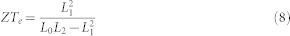


## Author Contributions

H.S. and S.S. have performed the calculations. H.S. and C.J.L. conceived the idea and wrote the paper.

## Supplementary Material

Supplementary Information Supplementary information

## References

[b1] Balandin A. A. Thermal properties of graphene and nanostructured carbon materials. Nat. Mater. 10, 569–581 (2011). 21778997 10.1038/nmat3064

[b2] Nika D. L. & Balandin A. A. Two-dimensional phonon transport in graphene. J. Phys.: Condens. Mat. 24, 233203 (2012). 10.1088/0953-8984/24/23/233203 22562955

[b3] Karamitaheri H., Neophytou N., Pourfath M., Faez R. & Kosina H. Engineering enhanced thermoelectric properties in zigzag graphene nanoribbons. J. Appl. Phys. 111, 054501 (2012).

[b4] Karamitaheri H., Pourfath M., Faez R. & Kosina H. Geometrical effects on the thermoelectric properties of ballistic graphene antidot lattices. J. Appl. Phys. 110, 054506 (2011).

[b5] Nakano H. *et al.* Soft synthesis of single-crystal silicon monolayer sheets. Angew. Chem. Int. Ed. 45, 6303–6306 (2006). 10.1002/anie.200600321 16941712

[b6] Cahangirov S., Topsakal M., Aktürk E., Şahin H. & Ciraci S. Two- and One-Dimensional Honeycomb Structures of Silicon and Germanium. Phys. Rev. Lett. 102, 236804 (2009). 19658958 10.1103/PhysRevLett.102.236804

[b7] Vogt P. *et al.* Silicene: Compelling Experimental Evidence for Graphenelike Two-Dimensional Silicon. Phys. Rev. Lett. 108, 155501 (2012). 22587265 10.1103/PhysRevLett.108.155501

[b8] Kim J., Fischetti M. V. & Aboud S. Structural, electronic, and transport properties of silicane nanoribbons. Phys. Rev. B. 86, 205323 (2012).

[b9] Kamal C., Chakrabarti A., Banerjee A. & Deb S. Silicene Beyond Mono-layers-Different Stacking Configurations And Their Properties. J. Phys.: Condens. Mat. 25, 085508 (2013). 10.1088/0953-8984/25/8/085508 23370369

[b10] Sadeghi H., Bailey S. & Lambert C. J. Silicene-based DNA nucleobase sensing. Appl. Phys. Lett. 104, 103104 (2014).

[b11] Huang S. T., Kang W. & Yang L. Electronic structure and quasiparticle bandgap of silicene structures. Appl. Phys. Lett. 102 (2013).

[b12] Sadeghi H. Electrical Transport Model of Silicene as a Channel of Field Effect Transistor. J. Nanosci. Nanotechnol. 14, 4178–4184 (2014). 24738367 10.1166/jnn.2014.8914

[b13] Jamgotchian H. *et al.* Growth of silicene layers on Ag (111): unexpected effect of the substrate temperature. J. Phys.: Condens. Mat. 24, 172001 (2012). 10.1088/0953-8984/24/17/172001 22487603

[b14] Feng B. *et al.* Evidence of Silicene in Honeycomb Structures of Silicon on Ag(111). Nano Lett. 12, 3507–3511 (2012). 22658061 10.1021/nl301047g

[b15] Enriquez H., Vizzini S., Kara A., Lalmi B. & Oughaddou H. Silicene structures on silver surfaces. J. Phys.: Condens. Mat. 24, 314211 (2012). 10.1088/0953-8984/24/31/314211 22820837

[b16] Lalmi B. *et al.* Epitaxial growth of a silicene sheet. Appl. Phys. Lett. 97, 223109 (2010).

[b17] Aufray B. *et al.* Graphene-like silicon nanoribbons on Ag(110): A possible formation of silicene. Appl. Phys. Lett. 96, 183102 (2010).

[b18] Chen L. *et al.* Evidence for Dirac Fermions in a Honeycomb Lattice Based on Silicon. Phys. Rev. Lett. 109, 056804 (2012). 23006197 10.1103/PhysRevLett.109.056804

[b19] Padova P. D. *et al.* Evidence of Dirac fermions in multilayer silicene. Appl. Phys. Lett. 102, 163106 (2013).

[b20] Molle A. *et al.* Hindering the Oxidation of Silicene with Non-Reactive Encapsulation. Adv. Funct. Mater. 23, 4340–4344 (2013).

[b21] Feng B. *et al.* Observation of Dirac cone warping and chirality effects in silicene. Acs. Nano. 7, 9049–9054 (2013). 24003914 10.1021/nn403661h

[b22] Chen L., Feng B. & Wu K. Observation of a possible superconducting gap in silicene on Ag(111) surface. Appl. Phys. Lett. 102, 081602 (2013).

[b23] Avila J. *et al.* Presence of gapped silicene-derived band in the prototypical (3 × 3) silicene phase on silver (111) surfaces. J. Phys.: Condens. Mat. 25, 262001 (2013). 10.1088/0953-8984/25/26/262001 23759650

[b24] Chen L., Feng B. & Wu K. Observation of superconductivity in silicene. *arXiv:1301.1431* (2013).

[b25] Lin C.-L. *et al.* Structure of silicene grown on Ag (111). Appl. Phys. Express. 5, 045802 (2012).

[b26] Tchalal M. R. *et al.* Formation of one-dimensional self-assembled silicon nanoribbons on Au (110)-(2×1). Appl. Phys. Lett. 102, 083107 (2013).

[b27] Meng L. *et al.* Buckled Silicene Formation on Ir(111). Nano Lett. 13, 685–690 (2013). 23330602 10.1021/nl304347w

[b28] Fleurence A. *et al.* Experimental Evidence for Epitaxial Silicene on Diboride Thin Films. Phys. Rev. Lett. 108, 245501 (2012). 23004288 10.1103/PhysRevLett.108.245501

[b29] Friedlein R., Fleurence A., Sadowski J. T. & Yamada-Takamura Y. Tuning of silicene-substrate interactions with potassium adsorption. Appl. Phys. Lett. 102, 221603 (2013).

[b30] Kokott S., Pflugradt P., Matthes L. & Bechstedt F. Nonmetallic substrates for growth of silicene: an ab initio prediction. J. Phys.: Condens. Mat. 26, 185002 (2014). 10.1088/0953-8984/26/18/185002 24728001

[b31] Zhang X. *et al.* Thermal conductivity of silicene calculated using an optimized Stillinger-Weber potential. Phys. Rev. B. 89, 054310 (2014).

[b32] Pan L. *et al.* Thermoelectric properties of armchair and zigzag silicene nanoribbons. Phys. Chem. Chem. Phys. 14, 13588–13593 (2012). 22965156 10.1039/c2cp42645e

[b33] Zberecki K., Wierzbicki M., Barnaś J. & Swirkowicz R. Thermoelectric effects in silicene nanoribbons. Phys. Rev. B. 88, 115404 (2013).

[b34] Yang K., Cahangirov S., Cantarero A., Rubio A. & D'Agosta R. Thermoelectric properties of atomically thin silicene and germanene nanostructures. Phys. Rev. B. 89, 125403 (2014).

[b35] Xie H., Hu M. & Bao H. Thermal conductivity of silicene from first-principles. Appl. Phys. Lett. 104, 131906 (2014).

[b36] Gu X. & Yang R. First-Principles Prediction of Phononic Thermal Conductivity of Silicene: a Comparison with Graphene. arXiv preprint *arXiv:1404.2874* (2014).

[b37] Bo L. *et al.* Thermal conductivity of silicene nanosheets and the effect of isotopic doping. J. Phys. D: Appl. Phys. 47, 165301 (2014).

[b38] Mingo N. & Broido D. A. Thermoelectric power factor of nanoporous semiconductors. J. Appl. Phys. 101, 014322 (2007).

[b39] Song D. W. *et al.* Thermal conductivity of nanoporous bismuth thin films. Appl. Phys. Lett. 84, 1883–1885 (2004).

[b40] Lee J.-H. & Grossman J. C. Thermoelectric properties of nanoporous Ge. Appl. Phys. Lett. 95, 013106 (2009).

[b41] Hu J., Ruan X. & Chen Y. P. Thermal Conductivity and Thermal Rectification in Graphene Nanoribbons: A Molecular Dynamics Study. Nano Lett. 9, 2730–2735 (2009). 19499898 10.1021/nl901231s

[b42] Chang P.-H. & Nikolic B. K. Edge currents and nanopore arrays in zigzag and chiral graphene nanoribbons as a route toward high-ZT thermoelectrics. Phys. Rev. B. 86 (2012).

[b43] Mortazavi B., Potschke M. & Cuniberti G. Multiscale modeling of thermal conductivity of polycrystalline graphene sheets. Nanoscale 6, 3344–3352 (2014). 24518878 10.1039/c3nr06388g

[b44] Zhang Y., Xu G., Han F., Wang Z. & Ge C. Preparation and Thermoelectric Properties of Nanoporous Bi2Te3-Based Alloys. J. Electron. Mater. 39, 1741–1745 (2010).

[b45] Lee H. *et al.* Effects of nanoscale porosity on thermoelectric properties of SiGe. J. Appl. Phys. 107, 094308 (2010).

[b46] Lee J.-H., Galli G. A. & Grossman J. C. Nanoporous Si as an Efficient Thermoelectric Material. Nano Lett. 8, 3750–3754 (2008). 18947211 10.1021/nl802045f

[b47] Ding Y. & Ni J. Electronic structures of silicon nanoribbons. Appl. Phys. Lett. 95, 083115 (2009).

[b48] Song Y. L., Zhang Y., Zhang J. M. & Lu D. B. Effects of the edge shape and the width on the structural and electronic properties of silicene nanoribbons. Appl. Surf. Sci. 256, 6313–6317 (2010).

[b49] Ding Y. & Wang Y. Electronic structures of zigzag silicene nanoribbons with asymmetric sp^2^−sp^3^ edges. Appl. Phys. Lett. 102, 143115 (2013).

[b50] Ezawa M. & Nagaosa N. Interference of Topologically Protected Edge States in Silicene Nanoribbons. Phys. Rev. B 88, 121401(R) (2013).

[b51] Nakada K., Fujita M., Dresselhaus G. & Dresselhaus M. S. Edge state in graphene ribbons: Nanometer size effect and edge shape dependence. Phys. Rev. B. 54, 17954–17961 (1996). 10.1103/physrevb.54.17954 9985930

[b52] Kobayashi Y., Fukui K.-i., Enoki T., Kusakabe K. & Kaburagi Y. Observation of zigzag and armchair edges of graphite using scanning tunneling microscopy and spectroscopy. Phys. Rev. B. 71, 193406 (2005).

[b53] Niimi Y. *et al.* Scanning tunneling microscopy and spectroscopy of the electronic local density of states of graphite surfaces near monoatomic step edges. Phys. Rev. B. 73, 085421 (2006).

[b54] Lahiri J., Lin Y., Bozkurt P., Oleynik I. I. & Batzill M. An extended defect in graphene as a metallic wire. Nat. Nano. 5, 326–329 (2010). 10.1038/nnano.2010.53 20348912

[b55] Anderson P., Lee P. & Saitoh M. Remarks on giant conductivity in TTF-TCNQ. Solid State Commun. 13, 595–598 (1973).

[b56] Soler J. M. *et al.* The SIESTA method for ab initio order- N materials simulation. J. Phys.: Condens. Mat. 14, 2745 (2002).

[b57] Perdew J. P., Burke K. & Ernzerhof M. Generalized Gradient Approximation Made Simple. Phys. Rev. Lett. 77, 3865–3868 (1996). 10062328 10.1103/PhysRevLett.77.3865

[b58] Berland K. & Hyldgaard P. Exchange functional that tests the robustness of the plasmon description of the van der Waals density functional. Phys. Rev. B. 89, 035412 (2014).

[b59] Ferrer J. *et al.* GOLLUM: a next-generation simulation tool for electron, thermal and spin transport. New J Phys 16, 093029 (2014).

