# Supplementary material for: Enhanced Thermoelectric Efficiency of Porous Silicene Nanoribbons
Source: Sci Rep. 2015 Mar 30;5:9514. doi: 10.1038/srep09514 (PMC4377624; doi:10.1038/srep09514)
Supplement: Supplementary Information [file srep09514-s1.pdf]

Supplementary Information

Enhanced Thermoelectric Efficiency of Porous Silicene Nanoribbons

Hatef Sadeghi*, Sara Sangtarash and Colin J Lambert†
Quantum Technology Centre, Lancaster University, LA1 4YB Lancaster, UK

Effect of varying the Fermi energy in doped silicene based structures: Fig. S1 shows the variation with E_F of ZT_e, κ_e and S in doped and un-doped silicene nanopores. Around the charge neutrality point (E_F=0), the slope of ZT_e for doped structures are high meaning that further small changes in the Fermi energy could improve the ZT_e significantly. As it is clear from inset of fig S1, TCNQ doped silicene shifts the Fermi energy to the right, as expected for a p-type dopant and TTF doping shifts the Fermi energy to the left, as expected for n-type doping. These shifts are in the range of 1-5 meV.

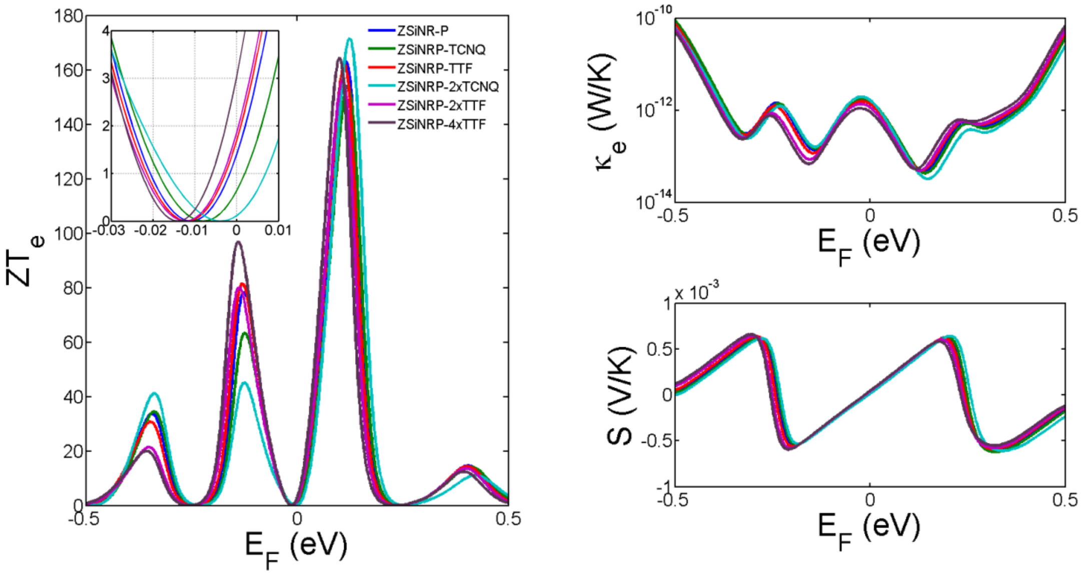

Figure S1: The variation of room-temperature values of ZT_e, S and κ as a function of E_F monolayer silicene nanopore (ZSiNR-P) with zigzag silicene ribbon lead, monolayer silicene nanopore with TCNQ functionalized zigzag silicene ribbon lead (ZSiNRP-TCNQ) and monolayer silicene nanopore with TTF functionalized zigzag silicene ribbon lead (ZSiNRP-TTF).

* *h.sadeghi@lancaster.ac.uk*
† *c.lambert@lancaster.ac.uk*

Figs 2a and 4a in more detail.

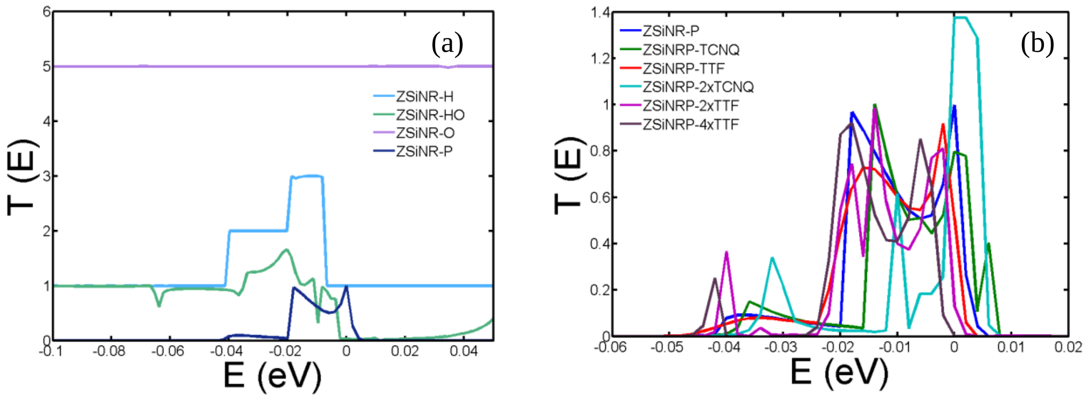

Figure S2: The transmission just around Fermi energy for (a) fig. 2a , (b) fig. 5a

The effect of a finite background (ie non-zero *a*) in the model T(E): For the model *T(E)* corresponding to smono-h (a=1, b=3) cases, the following figure shows that the presence of a non-zero background suppresses the achievable values of *ZT*.

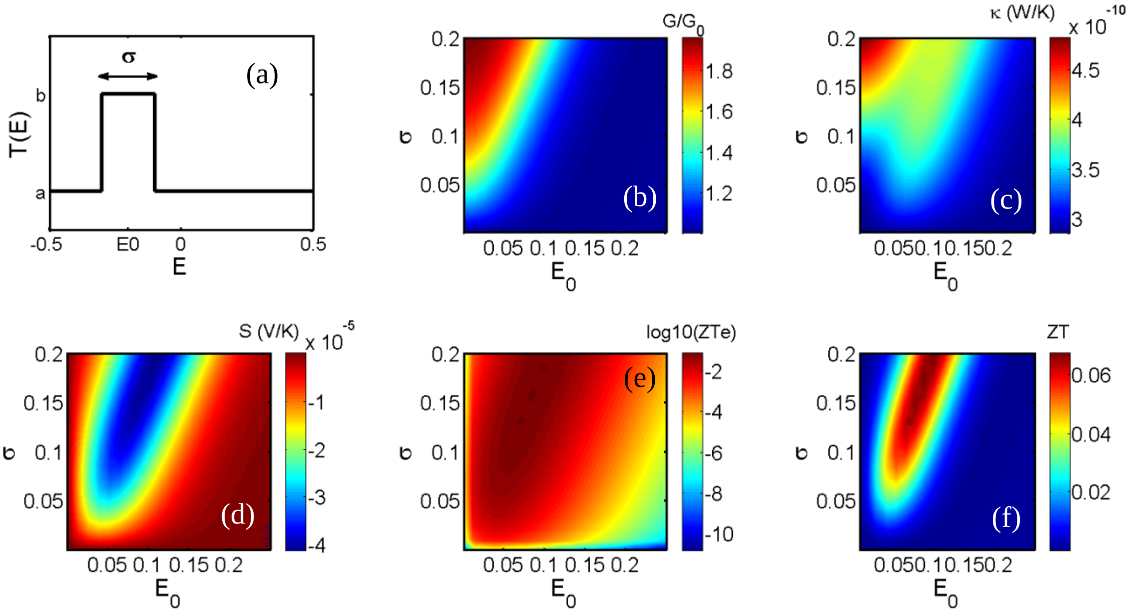

Figure S3: (a) Transmission coefficient with the width of σ and mean of E_0 and a=1, b=2 and corresponding (b) conductance (G/G_0), (c) electronic thermal conductance (κ_e), (d) Seebeck coefficient (S), (e) logarithm of electronic ZT (ZT_e) and (f) total ZT as a function of σ and E_0 in room temperature.

For the case of smono-o (a=5, b=5) *T(E)* energy independent within the simple model of fig S3a and *S* and *ZT* are zero.

The effect of using vdW density functional in the DFT calculation: Ideally one should include van der Waals interactions in the density functional as described in our recent article: [1]. However this is rather

expensive, which is why many papers in the literature use the GGA-PBE functional to study the effect of adorbants such as TTF and TCNQ on graphene and carbon nanotubes. Examples include: [2-8].

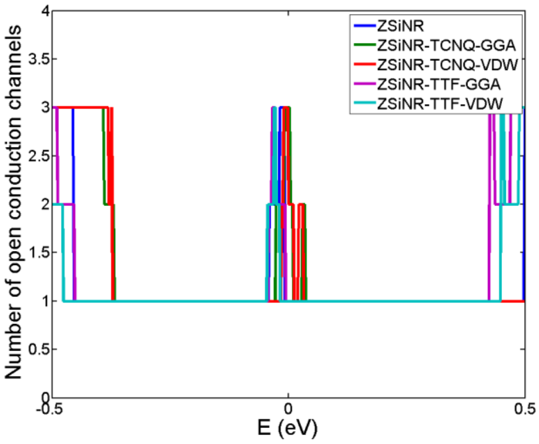

Figure S4: Number of open conduction channels in the zigzag silicene nanoribbon (ZSiNR), with and without dopants with GGA-PBE and VDW-BH functionals

To demonstrate the effect of the vdW functional, we performed two calculations with one layer of TTF and TCNQ on top of the silicene nanoribbons (ZSiNR). The vdW calculation is very expensive so we designed a demonstrator calculation to see if the shift in the Fermi energy due to TTF and TCNQ (obtained using the GGA-PBE functional) is reproduced by the vdW functional. Therefore we relaxed the TTF and TCNQ on top of the perfect ZSiNR with vdW functional. All other parameters are the same as those used in the calculation performed with GGA-PBE. The simulation result shows that the distance between the ZSiNR and dopant are in general smaller within vdW calculation as expected due to the additional vdW forces. Geometrically, the TTF and TCNQ are bent upward and downward on the ZSiNR. The distance between the dopant and ZSiNR are 3.49Å and 3.35Å in the middle and 3.63Å and 3.02Å in the edges of the TTF and TCNQ respectively.

The following figure shows the number of open channels in ZSiNR with and without dopant with vdW-BH and GGA-PBE functionals (see methods). It is apparent that in the vicinity of the Fermi energy, there are slight differences, but to within the accuracy of DFT and these are not significant.

Although vdW is needed to treat weak interactions, figure 6 shows that this is not needed to treat the electrostatic effects of adsorbates on silicene. In Figure 6, to achieve 43 and 87 % concentration, we have used double layer TTF on the silicene, meaning that the second additional layer has a distance of about 6.4Å, which is very far from the surface of the silicene and in the very weak coupling regime. Nevertheless, it is clear that the electrostatic effect on the silicene Fermi energy is captured.

SI References

1. Bailey, S., et al., *A study of planar anchor groups for graphene-based single-molecule electronics.* The Journal of chemical physics, 2014. **140**(5): p. 054708.
2. Cheng, J., et al., *Surface Plasmon Engineering in Graphene Functionalized with Organic Molecules: A Multiscale Theoretical Investigation.* Nano letters, 2013. **14**(1): p. 50-56.
3. Manna, A.K. and S.K. Pati, *Doping single-walled carbon nanotubes through molecular charge-transfer: a theoretical study.* Nanoscale, 2010. **2**(7): p. 1190-1195.
4. Tang, Q., Z. Zhou, and Z. Chen, *Molecular charge transfer: a simple and effective route to engineer the band structures of BN nanosheets and nanoribbons.* The Journal of Physical Chemistry C, 2011. **115**(38): p. 18531-18537.

5. Fraxedas, J., et al., *Modulation of surface charge transfer through competing long-range repulsive versus short-range attractive interactions.* The Journal of Physical Chemistry C, 2011. **115**(38): p. 18640-18648.
6. Bandyopadhyay, A., S.S. Yamijala, and S.K. Pati, *Tuning the electronic and optical properties of graphene and boron-nitride quantum dots by molecular charge-transfer interactions: a theoretical study.* Physical Chemistry Chemical Physics, 2013. **15**(33): p. 13881-13887.
7. Krukowski, S., P. Kempisty, and P. Strąk, *Fermi level influence on the adsorption at semiconductor surfaces—ab initio simulations.* Journal of Applied Physics, 2013. **114**(6): p. -.
8. Manna, A.K. and S.K. Pati, *Tuning the Electronic Structure of Graphene by Molecular Charge Transfer: A Computational Study.* Chemistry – An Asian Journal, 2009. **4**(6): p. 855-860.
